# Recent Developments in the Surgical Treatment of Bone Tumors and Their Impact on Quality of Life

**DOI:** 10.1155/2013/826432

**Published:** 2013-07-14

**Authors:** Andreas F. Mavrogenis, Ulrich Lenze, Hans Rechl, G. Douglas Letson, Pietro Ruggieri

**Affiliations:** ^1^Department of Orthopaedics, University of Bologna, Bologna, Italy; ^2^Klinikum rechts der Isar, Technical University of Munich, Munich, Germany; ^3^The Nuffield Department of Orthopaedics, Rheumatology and Musculoskeletal Science (NDORMS), University of Oxford, UK; ^4^Moffitt Cancer Center & Research Institute, 12902 Magnolia Drive Tampa, FL 33612, USA

The management of bone tumors has rapidly evolved over the last decades. Before the 1970s, amputation and arthrodesis were almost exclusively performed at the surgical theaters of tertiary tumor centers. Currently, with the evolutions in diagnostic imaging, surgical techniques, metallurgy, and adjuvant therapies, more than 90% of bone sarcomas patients are treated with limb salvage surgery. It is well documented in the related literature that limb salvage surgery does not compromise the survival of the patients. The main indication for limb salvage is the ability to obtain wide-margin (microscopically negative) surgical resection. A relative contraindication is local recurrence in a patient that previously had limb salvage surgery, except if the recurrence can be excised with wide margins. Various reconstruction options have been described following bone tumors resection with limb salvage, including megaprosthetic and biological reconstructions with allografts and vascularized bone autografts. However, what is the impact of bone tumors surgical resections and their reconstructions on the quality of life of tumor patients? This special issue tries to address the recent developments in the surgical treatment of bone tumors and their impact on quality of life. Expert authors in tumor surgery present their experience and knowledge on this subject.

The treatment of bone sarcomas is based on the tumor's biology and location and the patient's expectations [1]. The reconstruction options are technically demanding, may require lengthy treatment protocols, and may be associated with complications, which are not acceptable in cancer patients. These constraints have triggered a need for new therapeutic concepts to design and engineer structural and functional bone grafts. The goal is for long-term repair and optimum clinical outcome using techniques to replace tissues with inert biological devices such as tissue engineering constructs [2, 3]. The study of Dr. B. M. Holzapfel et al. is within this context. The authors discuss the implementation of tissue engineering concepts in treatment strategies of bone defects following bone tumor resection and outline their future prospects and possible application spectrum.

Massive bone allografts may be used as alternative to megaprosthetic reconstructions for bone defects after tumor resection. However, the rate of complications, namely, infection, fracture, and nonunion, may range up to 30%. Decreasing nonunion may minimize surgical exposure and permit earlier rehabilitation of the patients. While congruous osteotomy cuts are desirable, exact matching surfaces are rarely achieved using a freehand technique [4]. Computer-assisted navigation may provide for more accurate osteotomies resulting in more congruent allograft-host junctions, potentially decreasing nonunion rates [5, 6]. Dr. A. Lall et al. suggested that the limited contact achieved using standard freehand techniques may increase the rate of nonunion, while in contrast, computer-assisted navigation may increase the contact area and improve the rate of union. In their study, the authors quantified the average surface contact areas across simulated intraoperative osteotomies using both a free-hand and a computer-assisted navigation technique following application of a limited-contact dynamic compression plate. They found that using a freehand technique, contact areas of only 30% were obtained. However, using computer-assisted navigation the average contact area increased to more than 43%. Therefore, future development of an oncology software package and oncology-related navigation hardware may serve an important role in decreasing nonunion rates in limb salvage surgery and allograft reconstruction.

Peripheral dedifferentiated chondrosarcomas are rare high grade malignant connective tissue tumors [7]. The radiographic characteristics of these lesions have been reported in descriptive terms in limited series [8, 9]; however, objective quantification of their imaging characteristics has not been performed. Dr. E. R. Henderson et al. studied a clinical series of patients with peripheral dedifferentiated chondrosarcomas aiming to define imaging criteria to facilitate better recognition of these uncommon tumors. The authors found that the imaging characteristics described for central dedifferentiated chondrosarcomas are similar to peripheral tumors. They observed mineralization in all tumors except one, a preexisting exostosis in half cases and corticomedullary continuity in only 7% of cases, and no difference on the incidence of mineralization or other characteristics based on tumor location. The authors suggest that peripheral mineralization with a bimorphic pattern on CT scan and the presence of a soft-tissue mass should be considered worrisome for a peripheral dedifferentiated chondrosarcoma, particularly in the setting of multiple hereditary exostoses.

Chondrosarcoma is the most common malignant tumor of the foot, followed by Ewing's sarcoma and osteosarcoma [10]. A relatively long delay in diagnosis has been reported for tumors of the foot. Additionally, foot sarcomas rarely develop metastases; this may be attributed to a less aggressive behavior of bone tumors at the foot compared to similar histologies in other sites of the skeleton [11–13]. Dr. M. Brotzmann et al. studied a series of patients with sarcomas of the foot. They confirmed previous reports that sarcomas in this location show a distinct biological behavior compared to the same tumor at other skeletal sites; foot sarcomas grow slower and are less aggressive than those at other anatomical locations. Interestingly, a delayed time to diagnosis is observed; however, the prognosis is similar to other locations.

The pediatric skeleton is unique because of the growing physes and the smaller bones that complicate reconstructions following tumor resection. Biological reconstructions are considered the goal standard in this age group [14, 15]. Dr. L. Bellanova et al. describe a technique to decrease the resection margins in the tibia, ensuring that the margins are adequate. They used rapid prototyping and manufactured a patient-specific instrument as a guide for tumor resection and a second for the bone allograft osteotomy to adjust the allograft to fit the resection gap accurately. Histological sections of the resected specimens showed tumor free margins. The presented technique may improve the surgical accuracy and patient safety in surgical oncology.

We congratulate the authors for their important contributions to this special issue. We believe that this issue will inform the readers on the recent developments in the surgical treatment of bone tumors and their impact on the quality of life of these patients and will enhance the literature on the management of patients with bone sarcomas.

*Andreas F.*  *Mavrogenis* *Andreas F.*  *Mavrogenis* *Ulrich Lenze* *Ulrich Lenze* *Hans Rechl* *Hans Rechl* *G. Douglas Letson* *G. Douglas Letson* *Pietro Ruggieri* *Pietro Ruggieri*
